# Persistent geographic variations in availability and quality of nursing home care in the United States: 1996 to 2016

**DOI:** 10.1186/s12877-019-1117-z

**Published:** 2019-04-11

**Authors:** Yun Wang, Qiuli Zhang, Erica S. Spatz, Yan Gao, Sheila Eckenrode, Florence Johnson, Shih-Yieh Ho, Shuang Hu, Chao Xing, Harlan M. Krumholz

**Affiliations:** 1 Department of Biostatistics, Harvard T.H. Chan School of Public Health, Boston, MA USA; 2 Center for Outcomes Research and Evaluation, Yale-New Haven Hospital, 1 Church Street, Suite 200, New Haven, CT 06510 USA; 3 Usher Institute of Population Health Sciences & Informatics, University of Edinburgh, Edinburgh, UK; 4 National Clinical Research Center of Cardiovascular Diseases, State Key Laboratory of Cardiovascular Disease, Fuwai Hospital, National Center for Cardiovascular Diseases, Chinese Academy of Medical Sciences and Peking Union Medical College, Beijing, China; 5 Section of Cardiovascular Medicine, Department of Internal Medicine, Yale School of Medicine, New Haven, CT USA; 6 Qualidigm, Wethersfield, CT USA; 7 Department of Health Policy and Management, Yale School of Public Health, New Haven, CT USA

**Keywords:** Nursing home, Post-acute care, Quality of care, Geographic variation, Health services

## Abstract

**Background:**

Availability of nursing home care has declined and national efforts have been initiated to improve the quality of nursing home care in the U.S. Yet, data are limited on whether there are geographic variations in declines of availability and quality of nursing home care, and whether variations persist over time. We sought to assess geographic variation in availability and quality of nursing home care.

**Methods:**

Retrospective study using Medicaid/Medicare-certified nursing home data from the Centers for Medicare & Medicaid Services, 1996–2016. Outcomes were 1) availability of all nursing home care (1996–2016), measured by the number of Medicaid/Medicare-certified beds for a given county per 100,000 population aged ≥65 years, regardless of nursing home star rating; 2) availability of 5-star nursing home care, measured by the number of Medicaid/Medicare-certified beds provided by 5-star nursing homes; and 3) utilization of nursing home beds, defined as the rate of occupied Medicaid/Medicare-certified beds among the total Medicaid/Medicare-certified beds.

**Results:**

From 1999 to 2016, availability of all nursing home care declined from 4882 (standard deviation: 931) to 3480 (912) beds, per 100,000 population aged ≥65 years. Persistent geographic variation in availability of nursing home care was observed; the correlation coefficient of county-specific availabilities from 1996 to 2016 was 0.78 (95% CI 0.77–0.79). From 2011 to 2016, availability of 5-star nursing home beds increased from 658 (303) to 895 (661) per 100,000 population aged ≥65 years. The correlation coefficient for county-specific availabilities from 2011 to 2016 was 0.54 (95% CI 0.51–0.56). Availability and quality of nursing home care were not highly correlated. In 2016, the correlation coefficient for county-specific availabilities between all nursing home and 5-star nursing home beds was 0.33 (95% CI 0.30–0.36). From 1996 to 2016, the utilization of certified beds declined from 78.5 to 72.2%. This decline was consistent across all census divisions, but most pronounced in the Mountain division and less in the South-Atlantic division.

**Conclusion:**

We observed persistent geographic variations in availability and quality of nursing home care. Availability of all nursing home care declined but availability of 5-star nursing home care increased. Availability and quality of nursing home care were not highly correlated.

**Electronic supplementary material:**

The online version of this article (10.1186/s12877-019-1117-z) contains supplementary material, which is available to authorized users.

## Background

Nursing homes, which provide institutional care to patients who cannot to be cared for at home and need 24-h nursing supervision and/or extensive custodial care, play an important role for individuals needing such care, especially among older adults [1]. In the United States, there were more than 15,600 nursing homes with 1.7 million licensed beds providing care to 1.4 million residents in 2015 [2]. Among nursing home residents, approximately 65% are long-term residents who receive care under Medicaid and 13% are short-term residents who receive post-acute care under Medicare [3]. The proportion of Medicare residents has increased over time. Approximately 30% (1.5 million) of hospitalized Medicare beneficiaries are referred to nursing homes at hospital discharge [4]. Nursing home care is associated with shorter hospital stays and lower hospital costs and has been reported to improve patients’ health and quality of life, and lower rates of unplanned readmissions [5–12]. Given the importance of nursing homes, national efforts have been initiated to monitor and improve the quality of such care. The Centers for Medicare & Medicaid Services (CMS) launched the Five-Star Quality Rating System in 2008 to publicly report nursing home performance [13–15]. High star-rated nursing homes represent high quality of care and studies found that these nursing homes gain more residents [16–19].

The need for nursing home care may be expected to increase with the aging population in the United States. Nevertheless, two recent reports have found that the utilization of nursing home care has declined over time; between 2000 and 2010, the number of nursing home residents aged 65 years or older declined by 20%. The number of nursing homes declined in the same period and then stabilized in 2014 [20, 21]. Although many factors, including the increase in home care utilization and assisted living facilities, and reimbursement changes for Medicare and Medicaid, may impact this decline, it is also unknown whether there were geographic variations in the decline of availability of nursing home care, whether such variations persisted over time, whether there were geographic variations in quality of nursing home care, and whether there were geographic variations in the relationship between availability of care and quality of care. Additionally, the utilization of nursing home beds between nursing homes in states that participated in the CMS Money Follows the Person program and nursing homes in states that did not participate in that program is also of interest. The Money Follows the Person program focuses on developing home/community-based services designed to reduce the use of institutional care to improve patient satisfaction and rebalance Medicaid payments [22–24]. This program could be associated with geographic variation in utilization of nursing home care.

Accordingly, we used 1996–2016 national nursing home data from CMS [25, 26], which includes nursing home 5-star ratings*,* to assess geographic variations in the availability and quality of nursing home care. By linking the nursing home data with the U.S. census and Medicare information, we also evaluated county- and nursing home-specific characteristics associated with the care.

## Methods

### Study sample

Nursing home data consist of the name and characteristics of each Medicaid/Medicare-certified nursing home from 1996 to 2016, including the number of Medicaid/Medicare-certified beds, number of residents in these beds, and various nursing home characteristics. The 1996 data were the most complete and available earliest data at the national level, publicly available from Nursing Home Compare (https://healthdata.gov/dataset/nursing-home-compare-data). To ensure that the analysis was based on a relatively long period and accounted for the fact that nursing homes may have consolidated during the 21-year study period, we required all nursing homes to have at least 7 years of data for inclusion in the study. This restriction ensured that each nursing home contributed at least 33% of data points over the study period. The completed 5-star rating data were available from 2011, and we required nursing homes to have at least 5 years of data to be included in the analysis. This restriction ensures that each nursing home was evaluated based on a relatively long period.

### Outcomes

Our primary outcomes were the availability of all nursing home care and availability of 5-star nursing home care. The availability of all nursing home care was defined as the number of Medicaid/Medicare-certified nursing home beds available for a given county per 100,000 population aged ≥65 years, regardless of nursing home star rating. We selected the age group ≥65 years because the majority of nursing home residents are in this range. We obtained the estimated county-specific population data from the annual American Community Survey (https://www.census.gov/programs-surveys/acs/about.html), conducted by the U.S. Census Bureau. The availability of 5-star nursing home care was defined as the number of Medicaid/Medicare-certified nursing home beds available for a given county per 100,000 population aged ≥65 years, restricted to 5-star nursing homes. CMS uses its Five-Star Quality Rating system [13] to measure nursing home performance. The rating system includes 3 indicators: health inspections, staffing hours, and quality measures (online Additional file 1: Text S1); each of these indicators has its own star rating, ranging from 1 to 5, with 5 stars representing the top performance level for that indicator. The composite of these ratings provides an overall rating, ranging from 1 to 5 stars, which was used in this study. Additionally, we examined the trend in the utilization of nursing home beds, defined as the rate of the occupied Medicaid/Medicare-certified beds among the total Medicaid/Medicare-certified beds.

### Characteristics of nursing home and county

Nursing home characteristics included ownership (not-for-profit, yes/no), nursing home location (inside a hospital, yes/no), years of Medicaid or Medicare certification, nursing home size (certified beds <25th, 25th–75th, and > 75th percentiles), change in ownership (changed in last 12 months, yes/no), nursing home geographic location (rural, yes/no), and resident council (both resident and family, yes/no). County characteristics included Consumer Price Index-adjusted median income, proportions of non-Hispanic white, non-Hispanic-black, Hispanic, female, aged 65 years or older, under national poverty level, Supplemental Nutrition Assistance Program participants, households without a car and low access to stores, and seniors with low income and low access to stores. Health risk factors and lifestyle included prevalence of age-adjusted adult diabetes and obesity, number of recreation or fitness facilities per 1000 population, number of fast-food restaurants per 1000 population, and physical inactivity. All information was available from the U.S. Census Bureau and the Centers for Disease Control and Prevention websites. We also included the 2013 county-specific average age-sex-race-adjusted nursing home Medicare reimbursement per Medicare beneficiary residing in a given county, available from the Dartmouth Atlas (http://www.dartmouthatlas.org/tools/downloads.aspx#spending).

### Statistical analysis

To assess geographic variation in the availability of all nursing home care, we calculated county-specific availability for each county and then mapped the availability in 1996 and 2016, shading counties with a gradient from red to green (lowest availability in red to the highest in green). We divided states into 9 census divisions based on the U.S. census definition [27]. To assess whether geographic variation in availability persisted over time, we calculated a Pearson correlation coefficient of the availability between 1996 and 2016, weighted by county-specific population. We also fitted a mixed model with a Poisson link function and county-specific random intercepts to model the number of certified beds, regardless of nursing home rating, as a function of an ordinal time variable (time = 0 for 1996 and time = 21 for 2016), to assess the trend in availability of all nursing home care from 1996 through 2016, adjusted for county-specific characteristics. The risk ratio of the time variable represents the annual change in availability. We repeated these analyses to assess the geographic variation and trend in the availability of 5-star nursing home care, with restricted the data from 2011 to 2016.

To assess the relationship between availability of all nursing home care and availability of 5-star nursing home care, we modeled the availability of all nursing home care as a function of 5-star nursing home care, adjusted for county-specific characteristics and time and stratified by regions. To identify county-specific factors associated with the availability of all nursing home care, we restricted the study sample to 2016, the most recent year of the study period, and fitted a mixed model to regress the availability of all nursing home care as a function of county-specific characteristics. Using the 2016 data, we also assessed the nursing home characteristics associated with the 5-star rating (yes/no). We included a spherical covariance structure in all models to account for spatial autocorrelation and differences between counties and nursing homes. The county-specific population aged ≥65 years was used as an offset in models with a Poisson link function. Analyses were conducted using SAS version 9.4 64-bit (SAS Institute Inc., Cary, North Carolina).

## Results

### Study sample

The study sample included 17,875 unique Medicaid/Medicare-certified nursing homes over the 21-year study period. In 2016, the median (inter-quartile-range [IQR]) number of Medicaid/Medicare-certified years of a nursing home in business was 26 (20–36), 30.4% of nursing homes were not-for-profit ownership, 4.4% were in a rural area, and 93.4% were certified by both Medicaid and Medicare (online Additional file 1: Table S1).

### Geographic variation in availability of all nursing home care

**Fig. 1:**
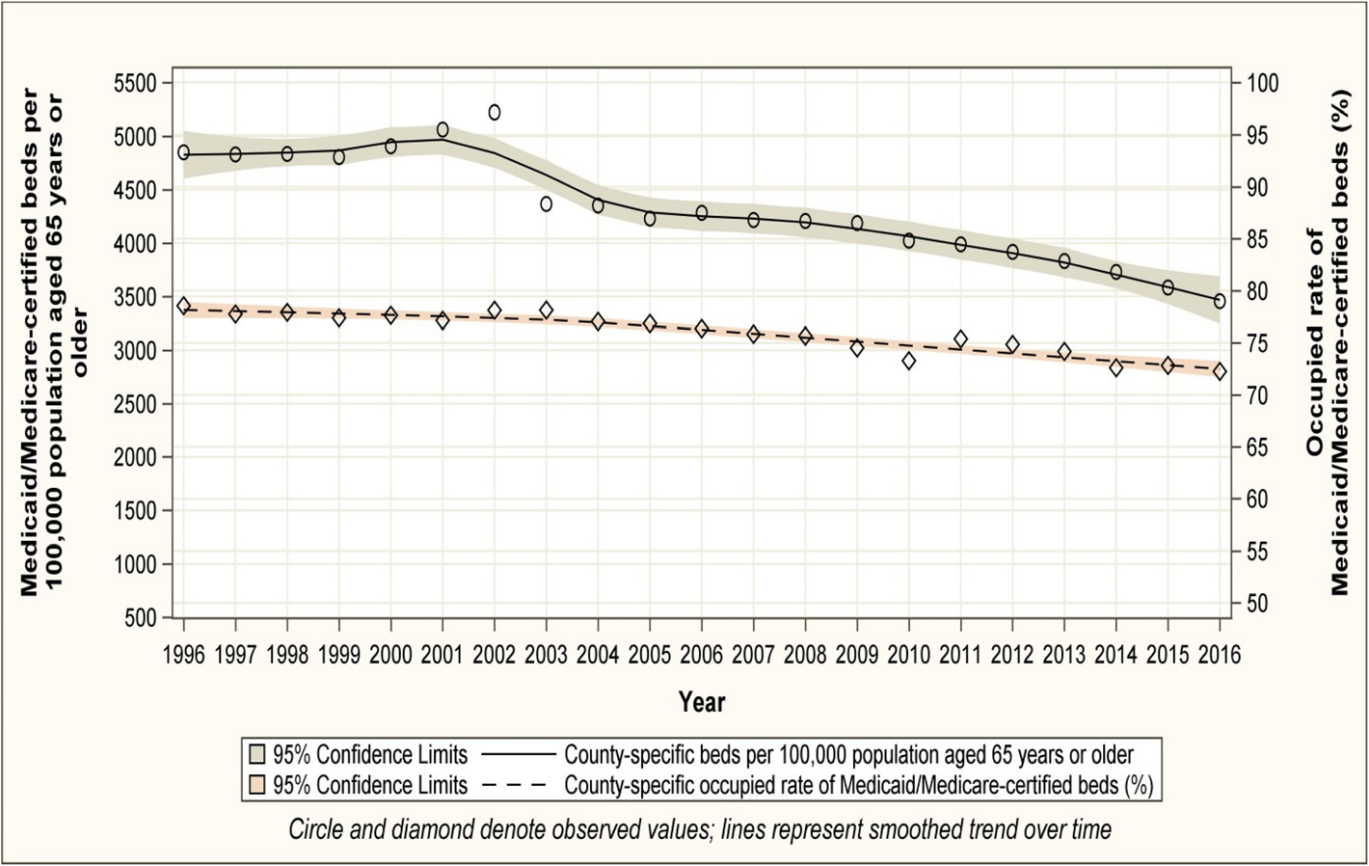
Trends in availability of nursing home care and utilization of Medicaid/Medicare-certified beds from 1996 to 2016. Availability of nursing home care is defined as the number of Medicaid/Medicare-certified beds available for a given county per 100,000 population aged 65 years or older, regardless of nursing home star rating. Utilization of nursing home beds is defined as the rate of the occupied Medicaid/Medicare-certified beds among the total Medicaid/Medicare-certified beds

**Fig. 2:**
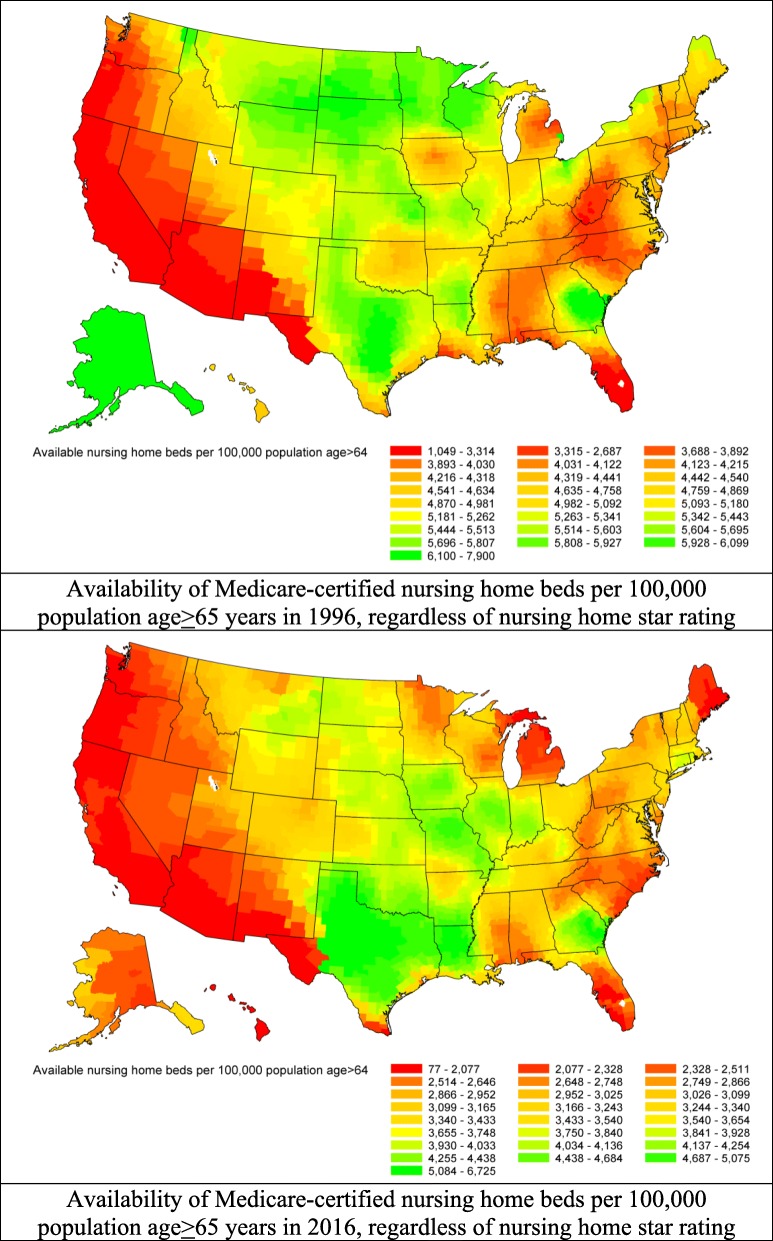
Geographic variations in the availability of Medicaid/Medicare-certified beds per 100,000 population aged 65 years or older by U.S. county. Top panel: availability in 1996; bottom panel: availability in 2016. Availability was mapped by shading counties with a gradient from red to green (lowest availability in red to highest availability in green)

**Fig. 3:**
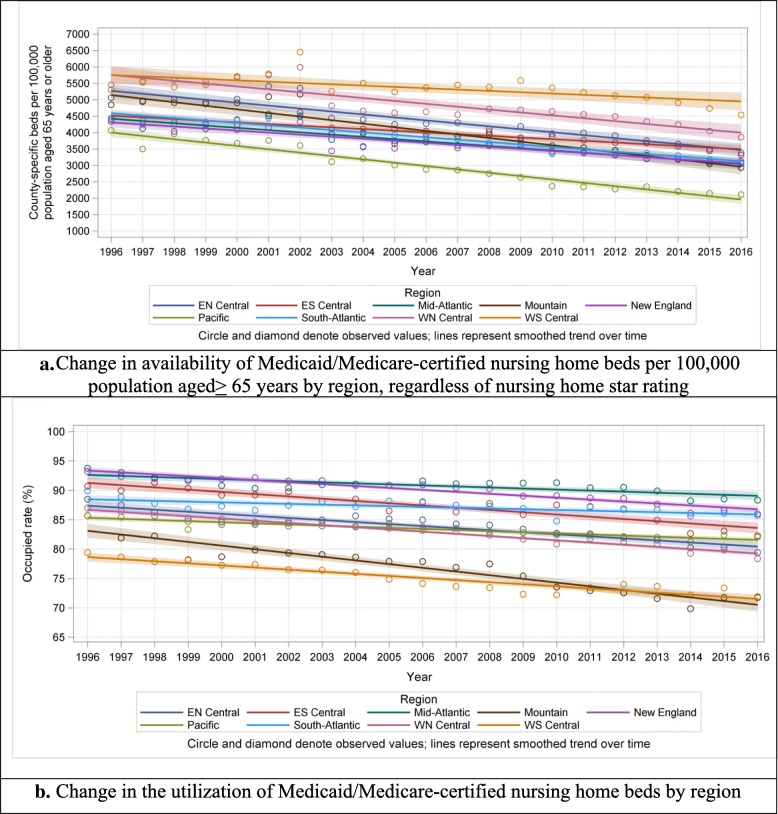
Regional variations in availability of Medicaid/Medicare-certified beds (top panel: **a**. Change in availability of Medicaid/Medicare-certified nursing home beds per 100,000 population aged >65 years by region, regardless of nursing home star rating) and utilization of Medicaid/Medicare-certified beds (bottom panel: **b**. Change in the utilization of Medicaid/Medicare-certified nursing home beds by region), 1996–2016

**Fig. 4:**
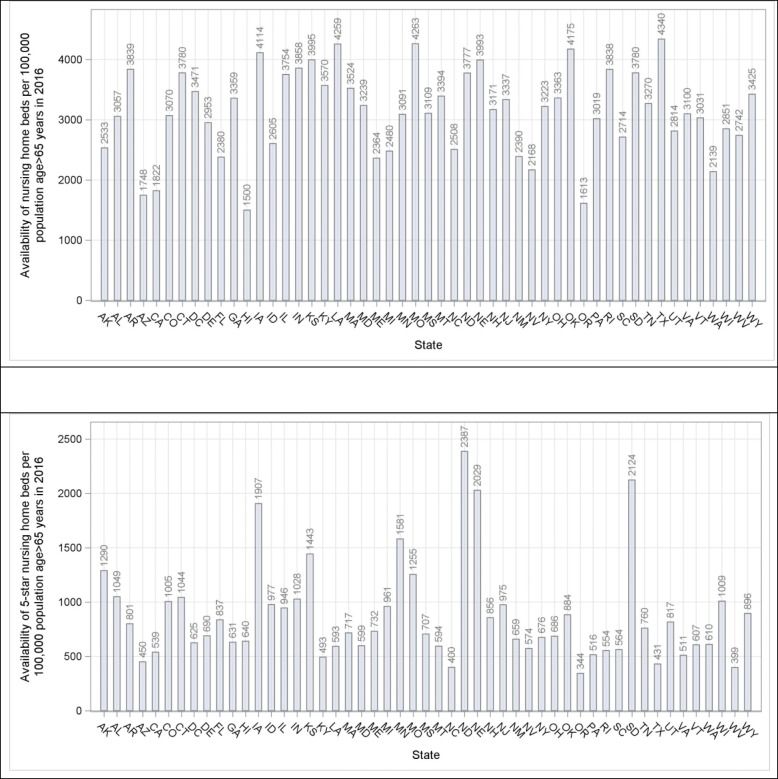
Availability of Medicaid/Medicare-certified beds per 100,000 population aged 65 years or older by U.S. state in 2016. Top panel: availability of Medicaid/Medicare-certified beds provided by all nursing homes, regardless of nursing home star rating; bottom panel: availability of Medicaid/Medicare-certified beds provided by 5-star nursing homes

Nationwide, the number (standard deviation [SD]) of Medicaid/Medicare-certified beds per 100,000 population aged ≥65 years declined from 4882 (931) in 1996 to 3480 (912) in 2016 (Fig. 1; online Additional file 1: Figure S1, top panel). Considerable geographic variation in the overall availability of nursing home care was observed. The county-specific number of Medicaid/Medicare-certified beds, per 100,000 population aged ≥65 years, ranged from 1049 to 7900 (difference 6851) in 1996 and from 70 to 6725 (difference 6655) in 2016 (Fig. 2). Availability in the West and East regions was lower than in the Central regions in both 1996 and 2016, and was higher in the West-South Central region (Fig. 2). The weighted Pearson correlation coefficient of county-specific availabilities between 1996 and 2016 was 0.78 (95% Confidence Interval (CI) 0.77–0.79), indicating that the geographic pattern of availability persisted over time. The county-specific characteristics adjusted annual decline in availability of all nursing home care, regardless of star rating, was 0.5% (95% CI 0.44–0.55). This decline was consistent across all census divisions but was most pronounced in the Pacific division, from 4064 beds per 100,000 population aged ≥65 years in 1996 to 2111 in 2016 (difference 1953), and least pronounced in the West-Central division, from 5296 per 100,000 population aged ≥65 years in 1996 to 4538 in 2016 (difference 758; Fig. 3, top panel). At the state level and in 2016, the 5 states with the highest mean (SD) availability of 5-star nursing home care were Texas (4340 [50]), Missouri (4263 [89]), Louisiana (4259 [593]), Oklahoma (4175 [107]), and Iowa (4114 [58] (Fig. 4, top panel). The 5 states with lowest availability were Hawaii (1500 [435]), Oregon (1613 [59]), Arizona (1748 [195]), California [1822 [61]), and Washington (2139 [98] (Fig. 4, top panel).

### Geographic variations in availability of 5-star nursing home care

**Fig. 5:**
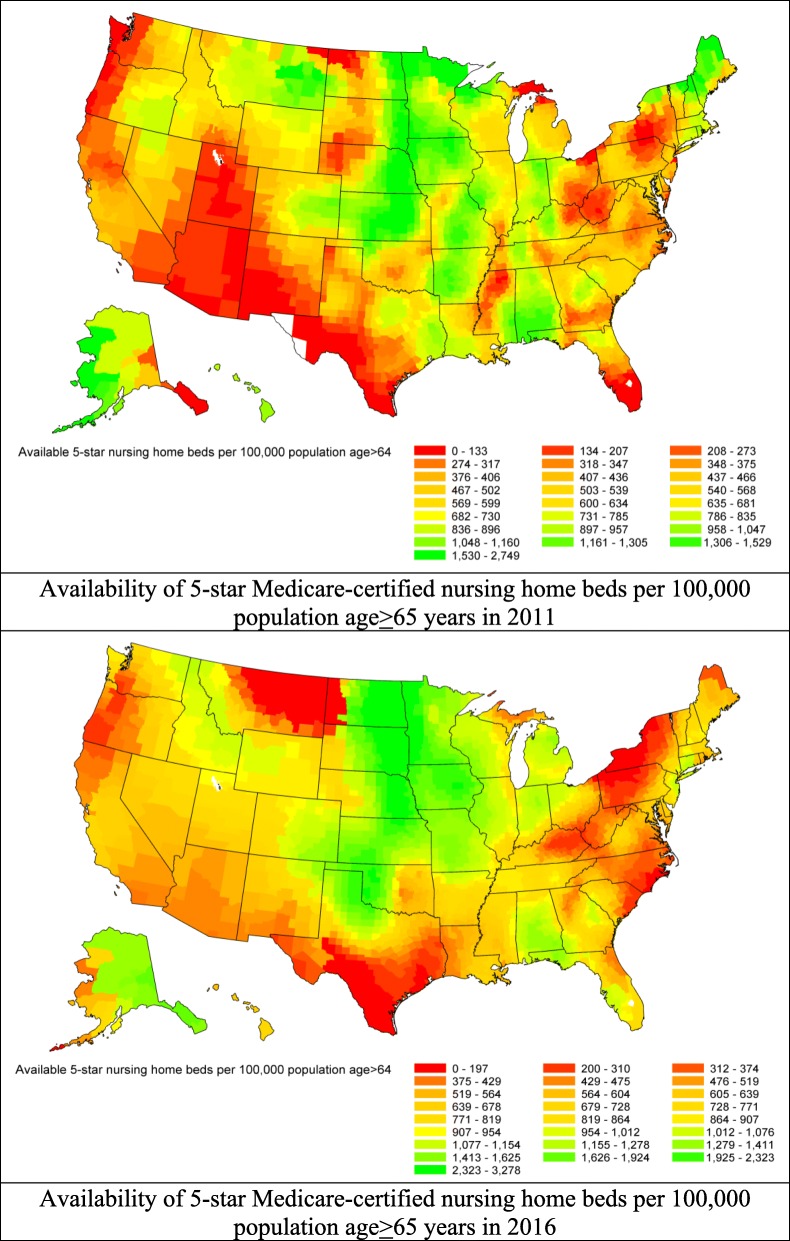
Geographic variations in the availability of 5-star Medicaid/Medicare-certified beds per 100,000 population aged 65 years or older by U.S. county. Top panel: availability in 2011; Bottom panel: availability in 2016. Availability was mapped by shading counties with a gradient from red to green (lowest availability in red to highest availability in green)

Between 2011 and 2016, the proportion of 5-star nursing homes increased from 16.2 to 24.0% (*p* < 0.01 for trend; online Additional file 1: Figure S2). The number (SD) of certified beds provided by 5-star nursing homes increased from 658 (303) in 2011 to 895 (661) in 2016, per 100,000 population aged ≥65 years (Fig. 5). Considerable geographic variation in the availability of 5-star nursing home care was observed. The county-specific number of Medicaid/Medicare-certified beds, per 100,000 population aged ≥65 years, provided by 5-star nursing homes ranged from 0 to 2749 (difference 2749) in 2011 and from 0 to 3278 (difference 3278) in 2016 (Fig. 5). The weighted Pearson correlation coefficient of the availabilities in 5-star nursing home care between 2011 and 2016 was 0.54 (95% CI 0.51–0.56), indicating moderately persistent geographic variation in availability of 5-star nursing home care. The county-specific characteristics adjusted annual increase in the availability of 5-star nursing home care was 9.5% (95% CI 8.41–10.64). At the state level and in 2016, the 5 states with the highest mean (SD) availability of 5-star nursing home care were North Dakota (2387 [88]), South Dakota (2124 [59]), Nebraska (2029 [61]), Iowa (1907 [27]), and Minnesota (1581 [33] (Fig. 4, bottom panel). The 5 states with lowest availability were Oregon (344 [10]), West Virginia (399 [7]), North Carolina (400 [5]), Texas (431 [5]), and Arizona (450 [48]) (Fig. 4, bottom panel).

### Relationship between availabilities of all nursing home and 5-star nursing home care

The relationship between availabilities of all nursing home, regardless of star rating, and 5-star nursing home care varied by regions (online Additional file 1: Figure S3). Accounting for county characteristics and time, the relationship between all and 5-star nursing home care was not significant for the East-North Central, Middle-Atlantic, Mountain, New England, and Pacific regions, while the South-Atlantic region was positive and the West-North Central was negative. In 2016, the weighted Pearson correlation coefficients for county-specific availabilities between all nursing home and 5-star nursing home care was 0.33 (95% CI 0.30–0.36), indicating that the availability of care and quality of care do not align. In 2016, only one state (Iowa) remained in the top 5 states with highest availability of nursing home care, regardless of star rating, as well as in the top 5 states with the highest availability of 5-star nursing home care.

### Characteristics associated with availability and quality of care

County geographic location is associated with availability of nursing home care. The availability for counties in the West South Central and West North Central regions was higher than for counties in other regions (online Additional file 1: Table S2). Counties with greater inactivity, more fast-food restaurants, more rural areas, and higher proportions of non-Hispanic white and non-Hispanic black populations were more likely to have higher availability of all nursing home care. Counties with higher income, higher proportions of people with *diabetes mellitus,* Supplemental Nutrition Assistance Program participants, and women were more likely to have lower availability of all nursing home care (online Additional file 1: Table S2). The age-sex-race-adjusted Medicare reimbursement dollars paid to a nursing home was associated with an increase in the availability of nursing home care (rate ratio 1.05, 95% CI 1.03–1.07). Nursing homes with a council joined by family and residents, with certified beds in the <75th percentile of the national average (130 beds in 2016) and with a high utilization of beds, were more likely to be 5-star. Nursing homes with for-profit ownership, located in a rural area, and in which ownership changed in the last 12 months were unlikely to be 5-star (online Additional file 1: Figure S4).

### Trends in utilization of nursing home beds

Between 1996 and 2016, the utilization of certified beds declined from 78.5 to 72.2% (Fig. 1; online Additional file 1: Figure S1, bottom panels). This decline was consistent across all census divisions, but was most pronounced in the Mountain division, from 86.7% in 1996 to 71.8% in 2016 (difference 14.9 percentage points), and less in the South-Atlantic division, from 89.9% in 1996 to 85.7% in 2016 (difference 4.2 percentage points; Fig. 3, bottom panel).

In 2016, 43 states plus the District of Columbia participated in the CMS Money Follows the Person program. There was no difference in the utilization of certified beds between nursing homes in states that participated in that program and nursing homes in states that did not participate (81.1% versus 80.7%, respectively, *p* = 0.703).

## Discussion

In this investigation, we observed notable and persistent geographic variations in the availability of nursing home care, the quality of nursing home care, and in the relationship between availability of nursing home care and 5-star nursing home care. Despite the decline in availability of nursing home care, the availability of 5-star nursing home care increased. However, the availability of nursing home care and 5-star nursing home care varied by regions. The correlation between nursing home availability and 5-star nursing home care was weak. In 2016, only one state remained in the top 5 states with highest availability of nursing home care as well as in the top 5 states with the highest availability of 5-star nursing home care. People in regions that have high availability of nursing home care may not receive high-quality care, suggesting that the competition does not drive improvement. This observation is aligned with our previous study on home health care [28].

We found that availability of nursing homes in the West and East regions was lower than in the Central region, which could reflect higher labor costs not covered by Medicare and Medicaid reimbursements. This finding also aligns with the service pattern in home health care [29].

According to CMS, nursing homes with high rating scores represent high quality of care for their residents. The number of 5-star homes is limited under the current CMS rating methodology, but every nursing home has an opportunity to be 5-star. Our study shows that those with the top performance are not concentrated in a particular area.

Additionally, we found that the CMS Money Follows the Person program was not associated with the decline in utilization of nursing homes, at least in 2016. There was no difference in the utilization of certified beds between nursing homes in states that participated in and nursing homes in states that did not participate in that program. One of the reasons could be that only a small proportion of residents were actually affected by this program. For example, from 2008 through 2016, 75,151 residents with chronic conditions and disabilities transitioned from institutions back into their communities [26]. Nevertheless, Robison et al. showed that the program yielded positive results in Connecticut [30].

Many factors could contribute to geographic variations in the availability and quality of nursing home care. The availability of care was high in the Central region, where many states do not have Certificate of Need programs designed to limit healthcare facility costs and facilitate coordinated planning of new services and facility construction [31]. Additionally, county-specific characteristics are associated with the geographic variation in nursing home care. For example, characteristics such as fast-food restaurants and physical inactivity were associated with greater nursing home use. We also found an association between registered nurses and nursing home ownership with the quality of service in the nursing homes, which complements and extends the findings of prior studies [32–35]. The negative association between the availability of nursing home beds and star rating may indicate that quality care is more achievable for small- or middle-sized nursing homes. We were not able to determine the underlying reason for why some nursing homes perform better than others. The Nursing Home Reform Act, a part of the Omnibus Budget Reconciliation Act of 1987, grants residents the right to organize and participate in a resident or family council [36, 37]. Compared with nursing homes with a resident-only council, nursing homes with a council that includes residents and their family members are more likely to be 5-star rated. It may be that residents’ family members monitor care and effectively improve care delivery [38, 39]. Conversely, a nursing home with more focus on quality may be more likely to invest in having such a council. The finding of a negative association between for-profit ownership of nursing homes and their performance extends prior studies [33, 34], but provides a more contemporary assessment at the national level.

We also found that there was an approximately 30% decline in availability of nursing home care from 1996 through 2016. One reason for such a decline may represent changes in market demand resulting from Medicaid and Medicare policy changes, including the adoption of prospective payment for Medicare-paid post-acute care as well as Medicaid-paid long-term home- and community-based care reforms [20, 40, 41]. These policy changes impact reimbursements for nursing homes. Moreover, even though Medicare beneficiaries were more likely to be referred to nursing home care or home health care at hospital discharge in recent years, the relative increase in referrals to home health care was greater than the increase in referrals to nursing home care [3]. A recent study found that the availability of home health care has increased over time [29]. As the aging of the U.S. population continues to increase, the market for in-home services is also likely to increase, as such care provides a potentially more desirable and cost-effective option.

Our study has several limitations. We focused on nursing home star ratings reported by CMS. Other measures that may also be associated with quality of care, such as use of antipsychotic medication, staff stability, or consistent assignment, were not available in our analysis. The criteria for stars might have changed over the study period, but we were unable to account for such a change. Our analysis was conducted at the nursing home-level, which was unable to separate the Medicare and Medicaid recipients nor identify skilled and non-skilled populations. Likewise, we were unable to assess trends in the length of stay in nursing homes, which would also be of interest. Since the scope of our data did not allow us to address these challenges, future studies are warranted.

## Conclusion

In conclusion, persistent geographic variations in availability and quality of nursing home care exist and the availability of nursing home care provided by all nursing homes has declined. Availability and quality of nursing home care were not highly correlated. More research is necessary to understand why some nursing homes perform better than others.

## Additional file

Additional file 1: Supplemental tables and figures referred to within the manuscript. (DOC 2921 kb)

## Abbreviations

CI: Confidence Interval
CMS: Centers for Medicare & Medicaid Services
IQR: Inter-quartile Range
SD: Standard Deviation
