# Supplementary material for: Persistent geographic variations in availability and quality of nursing home care in the United States: 1996 to 2016
Source: BMC Geriatr. 2019 Apr 11;19:103. doi: 10.1186/s12877-019-1117-z (PMC6460800; doi:10.1186/s12877-019-1117-z)
Supplement: Supplementary file 1 — Supplemental tables and figures referred to within the manuscript. (DOC 2921 kb) [file 12877_2019_1117_MOESM1_ESM.doc]

**Additional file**

**eText 1. Indicators of CMS home performance measurement.**

**eText 2. Characteristics of nursing home.**

**eTable 1. Nursing home characteristics and quality of measures 2011-2016 (nursing homes with at least 5 years of data).**

**eTable2. Factors associated with availability of all nursing home care based on 2016 data.**

**eFigure1. Changes in availability of nursing home care and occupied rate of Medicaid/Medicare-certified beds.**

**eFigure 2. Change in the coefficient of dispersion of star rating overall and by individual indicator. A decrease in the dispersion over time implies reduced heterogeneity in the star rating.**

**eFigure 3. Change in nursing home star rating 2011-2016.**

**eFigure 4. Relationship between the availabilities of all nursing home and 5-star nursing home care.**

**eFigure 5. Nursing home characteristics associated with being a 5-star rated nursing home in 2016.**

**eText 1. Indicators of CMS Home Performance Measurement.**

CMS measures nursing home performance with 3 indicators: 1) health inspections (the health inspection rating contains information from the last 3 years of onsite inspections, including both standard surveys and any complaint surveys), 2) staffing hours (information about the number of hours of care provided on average to each resident each day by nursing staff, including registered nurse, licensed practical nurse and licensed vocational nurse, certified nursing assistant, and physical therapist), and 3) quality measures (information on 11 different physical and clinical measures for nursing home residents).

**eText 2. Characteristics of Nursing Home.**

Nursing home characteristics include ownership (not-for-profit, yes/no), nursing home location (inside a hospital, yes/no), years of Medicaid or Medicare certification, nursing home size (certified beds <25th, 25th-75th, and >75th percentiles), change in ownership (changed in last 12 months, yes/no), nursing home geographic location (rural, yes/no), and resident council (both resident and family, yes/no). County characteristics included Consumer Price Index-adjusted median income, proportions of non-Hispanic white, non-Hispanic-black, Hispanic, female, aged 65 years or older, under national poverty level, Supplemental Nutrition Assistance Program participants, households without a car and low access to stores, and seniors with low income and low access to stores. Health risk factors and lifestyle included prevalence of age-adjusted adult diabetes and obesity, number of recreation or fitness facilities per 1000 population, number of fast-food restaurants per 1000 population, and physical inactivity. All information was available from the U.S. Census Bureau and the Centers for Disease Control and Prevention websites. We also included the 2013 county-specific average age-sex-race-adjusted nursing home Medicare reimbursement per Medicare beneficiary residing in a given county, available from the Dartmouth Atlas ([http://www.dartmouthatlas.org/tools/downloads.aspx#spending](http://www.dartmouthatlas.org/tools/downloads.aspx#spending)).

**eTable 1. Nursing home characteristics and quality of measures 2011-2016**

**(nursing homes with at least 5 years of data).**

|  | **Measure** | **2011** | **2012** | **2013** | **2014** | **2015** | **2016** |
| --- | --- | --- | --- | --- | --- | --- | --- |
| **Characteristics** | |  |  |  |  |  |  |
|  | Total number of included nursing homes, # | 15,498 | 15,331 | 15,185 | 15,072 | 14,936 | 14,822 |
|  | Years of Medicare certification, median (IQR) | 21 (15-31) | 22 (16-32) | 23 (17-33) | 24 (18-34) | 25 (19-35) | 26 (20-36) |
|  | Located inside a hospital, % | 6.2 | 6.0 | 5.7 | 5.4 | 5.3 | 5.0 |
|  | Ownership as not-for-profit, % | 30.9 | 30.7 | 30.3 | 29.2 | 30.8 | 30.4 |
|  | Ownership changed in last 12 months, % | 2.3 | 3.8 | 2.2 | 1.5 | 2.8 | 2.6 |
|  | Council with both resident and family, % | 30.4 | 28.5 | 26.74 | 25.6 | 24.1 | 22.9 |
|  | Located in a rural area, % | 4.6 | 4.6 | 4.6 | 4.5 | 4.5 | 4.4 |
|  | Medicaid-certified only, % | 3.5 | 3.2 | 3.1 | 2.8 | 2.6 | 2.4 |
|  | Medicare-certified only, % | 4.8 | 4.8 | 4.6 | 4.5 | 4.3 | 4.2 |
|  | Medicaid- and Medicare-certified, % | 91.7 | 92.0 | 92.3 | 92.7 | 93.1 | 93.4 |
|  | Medicaid- and/or Medicare-certified beds, median (IQR) | 100 (63-129) | 100 (64-128) | 100 (64-129) | 100 (64-128) | 100 (65-129) | 100 (66-130) |
|  | Certified-beds <25 percentile of all nursing homes, % | 50.5 | 50.2 | 50.6 | 50.5 | 50.5 | 51.0 |
|  | Certified-beds 25-75 percentile of all nursing homes, % | 24.8 | 24.8 | 24.62 | 25.8 | 24.7 | 24.9 |
|  | Certified-beds >75 percentile of all nursing homes, % | 24.7 | 25.0 | 24.82 | 23.7 | 24.8 | 24.1 |
| **Star rating** | |  |  |  |  |  |  |
|  | 5-star nursing homes, % | 15.6 | 20.5 | 21.7 | 23.8 | 23.7 | 24.0 |
|  | Overall, mean (SD) | 3.1 (1.3) | 3.2 (1.3) | 3.4 (1.3) | 3.3 (1.3) | 3.2 (1.4) | 3.2 (1.4) |
|  | Inspection, mean (SD) | 2.8 (1.3) | 2.8 (1.3) | 2.9 (1.3) | 2.9 (1.3) | 2.8 (1.3) | 2.8 (1.3) |
|  | Total staffing hours per resident day, mean (SD) | 3.2 (1.2) | 3.2 (1.2) | 3.3 (1.1) | 3.3 (1.1) | 3.2 (1.1) | 3.2 (1.2) |
|  | Quality measures, mean (SD) | 3.2 (1.2) | 3.7 (1.1) | 3.8 (1.0) | 3.7 (1.2) | 3.5 (1.4) | 3.5 (1.4) |
| IQR: inter-quartile range; SD: standard deviation | | | | | | | |

**eTable2. Factors associated with availability of all nursing home care based on 2016 data.**

| **Characteristics** | **Incidence Rate Ratio (95% Confidence Interval)*** | **P value** |
| --- | --- | --- |
| **Geographic Division** |  |  |
| South Atlantic | 0.58 (0.55-0.62) | < 0.001 |
| Middle Atlantic | 0.79 (0.73-0.85) | < 0.001 |
| New England | 0.80 (0.72-0.89) | < 0.001 |
| East North Central | 0.87 (0.82-0.93) | < 0.001 |
| East South Central | 0.69 (0.65-0.74) | < 0.001 |
| West North Central | 1.08 (1.02-1.16) | 0.02 |
| Mountain | 0.65 (0.60-0.71) | < 0.001 |
| Pacific  West South Central (reference) | 0.52 (0.48-0.57) | < 0.001 |
| **Sociodemographic** |  |  |
| Proportion female | 0.80 (0.74-0.86) | < 0.001 |
| Proportion non-Hispanic white | 1.51 (1.10-2.06) | 0.01 |
| Proportion non-Hispanic black | 2.13 (1.55-2.92) | < 0.001 |
| Proportion Hispanic | 1.18 (0.85-1.64) | 0.33 |
| Median income ($10,000) | 0.99 (0.99-1.00) | < 0.001 |
| Proportion in the Supplemental Nutrition Assistance Program | 0.75 (0.69-0.81) | < 0.001 |
| Rate of households without car and low access to stores | 1.02 (0.92-1.12) | 0.73 |
| Rate of seniors with low income and low access to stores | 1.77 (0.97-3.23) | 0.06 |
| **Health Risk Factors and Lifestyle** |  |  |
| Age-adjusted adult obesity rate | 0.87 (0.48-1.56) | 0.64 |
| Number of recreation or fitness facilities per 1000 population | 0.90 (0.68-1.18) | 0.44 |
| Number of fast-food restaurants per 1000 population | 1.30 (1.21-1.40) | < 0.001 |
| Age-adjusted physical inactivity rate | 17.63 (10.28-30.22) | < 0.001 |
| **Medicare Reimbursement** |  |  |
| Price-age-sex-race-adjusted Medicare hospital and skilled nursing facility reimbursements per Medicare beneficiary in a given county ($) | 1.05 (1.03-1.07) | < 0.001 |
| *****An incidence rate ratio <1 indicates that an increase in that county-specific characteristic is associated with a decrease in the availability of home health care. Similarly, an incidence rate ratio >1 indicates that an increase in that county-specific characteristic is associated with an increase in the availability of home health care. | | |

**eFigure1. Changes in availability of nursing home care and occupied rate**

**of Medicaid/Medicare-certified beds.**

| **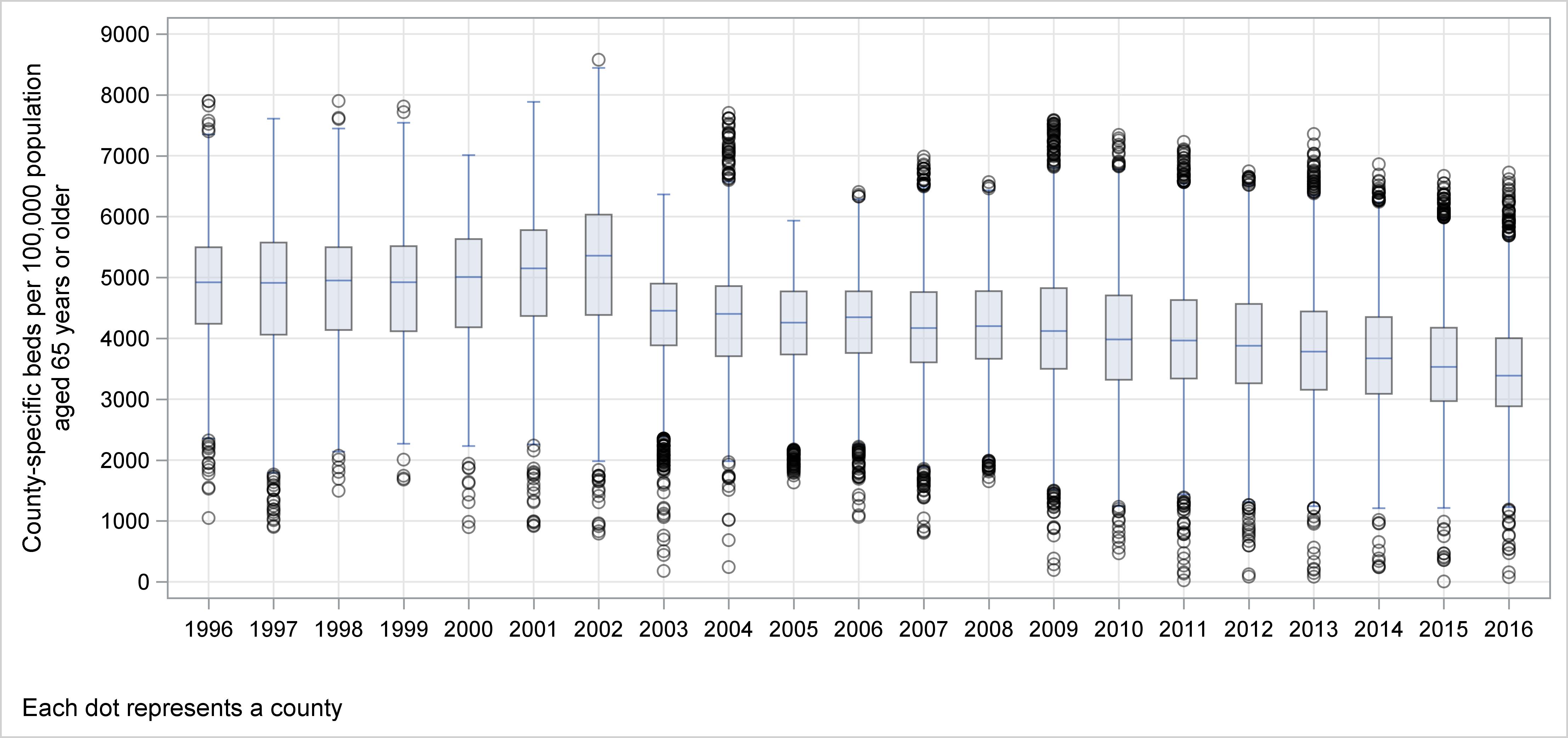** |
| --- |
| a. All nursing home care availability 1996-2016 |
| **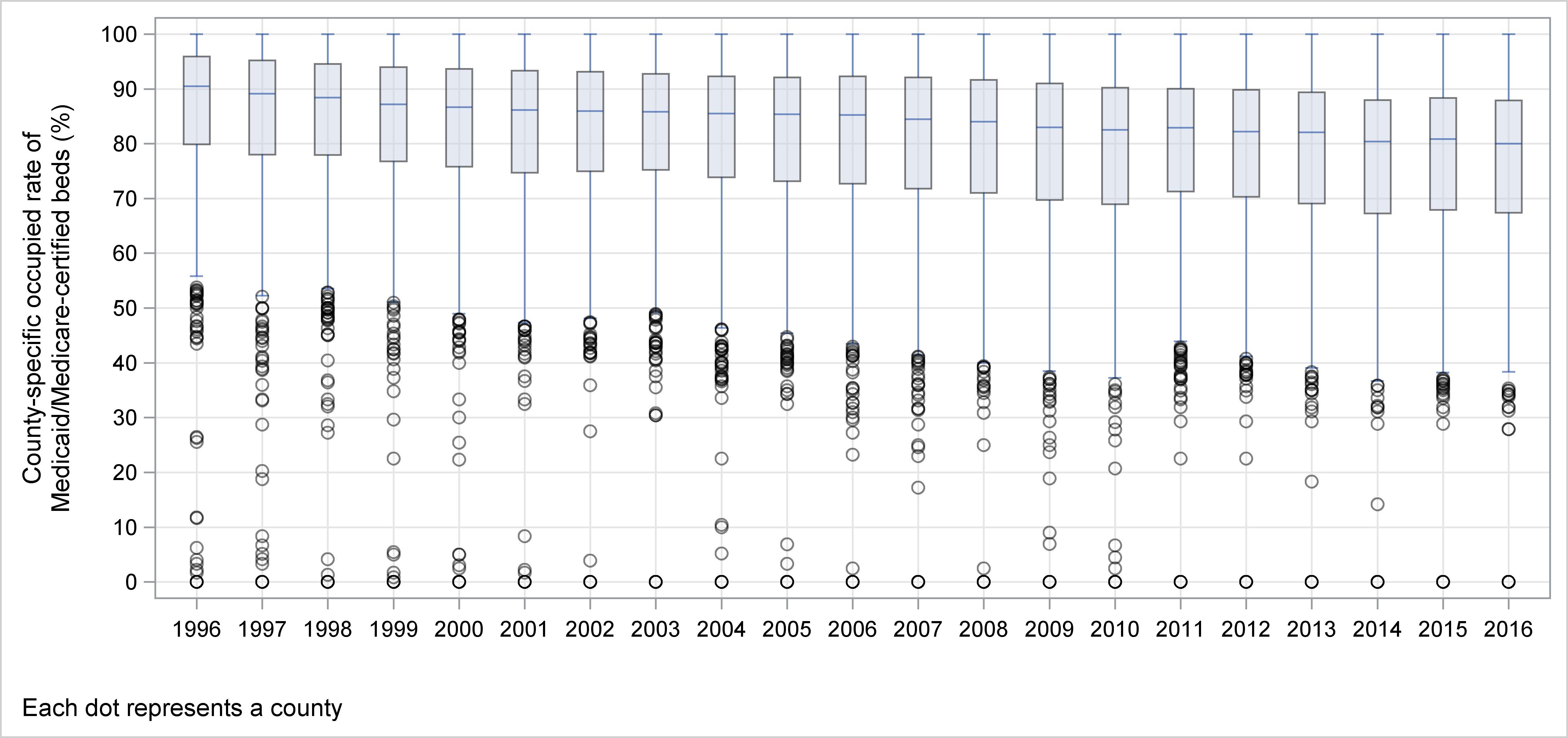** |
| b. Utilization of Medicaid/Medicare-certified beds 1996-2016 |

Box and whisker plots of the distributions of county-specific nursing home beds per 100,000 population aged 65 years or older and the utilization of Medicaid/Medicare-certified beds. The length of the box represents the inter-quartile range (IQR), the horizontal line in the box interior represents the median, the whiskers represent the 1.5 IQR of the 25th quartile or 1.5 IQR of the 75th quartile, and the dots represent outliers.

**eFigure 2. Change in the coefficient of dispersion of star rating overall and by individual indicator. A decrease in the dispersion over time implies reduced heterogeneity in the star rating.**

**
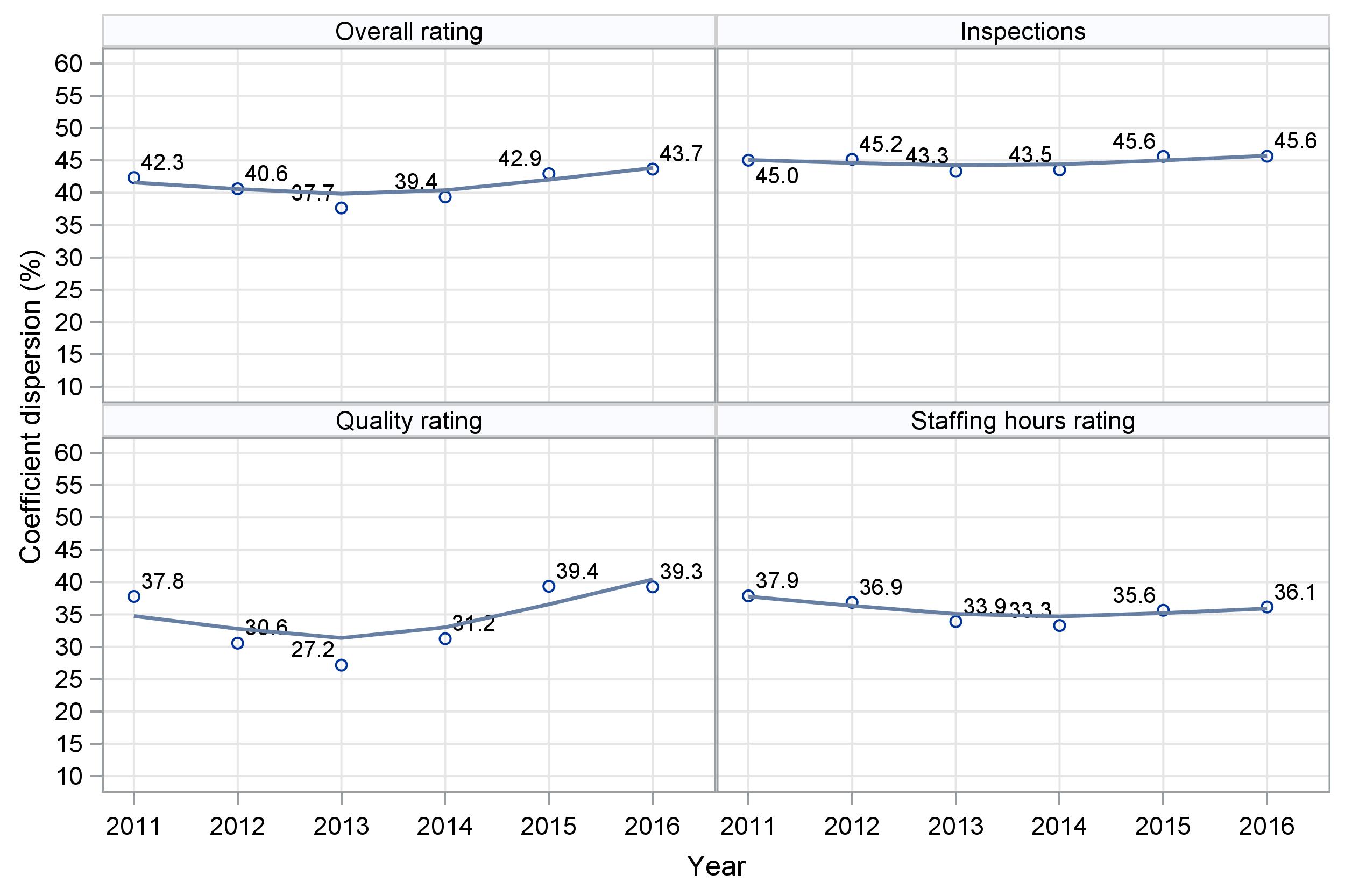
**

**eFigure 3. Change in nursing home star rating 2011-2016.**

**
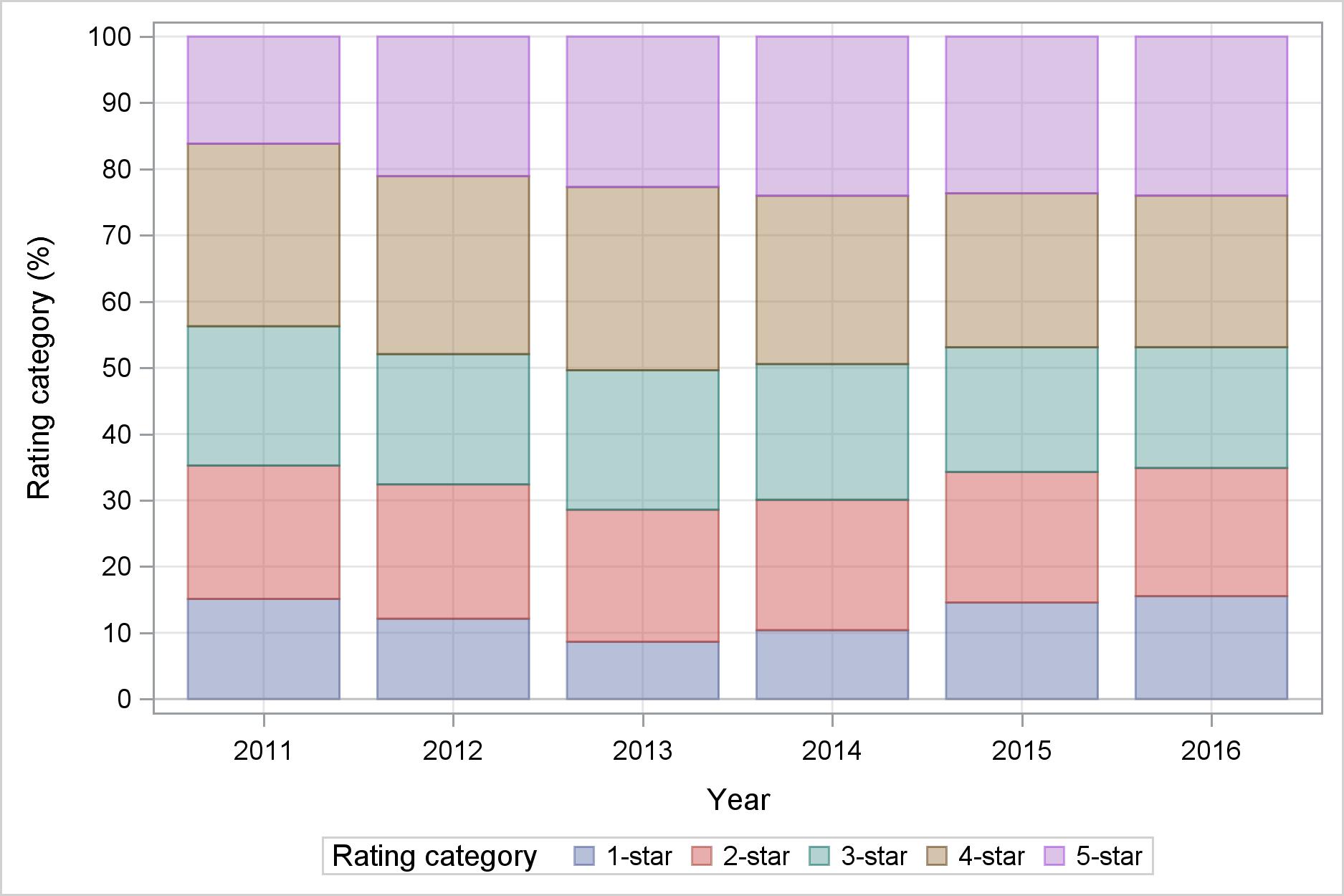
**

**eFigure 4. Relationship between the availabilities of all nursing home**

**and 5-star nursing home care.**

**
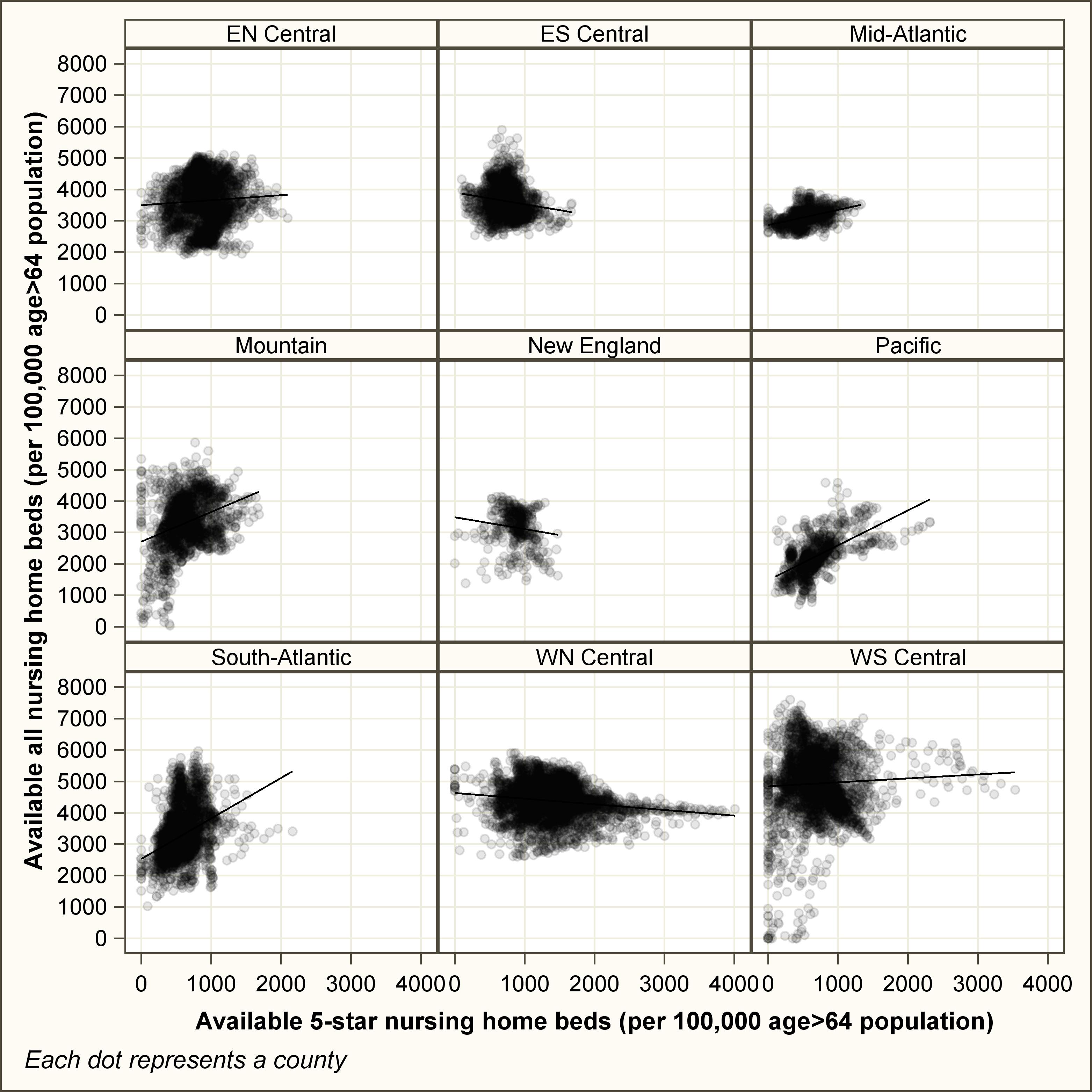
**

**eFigure 5. Nursing home characteristics associated with being a 5-star rated nursing home in 2016.**

**
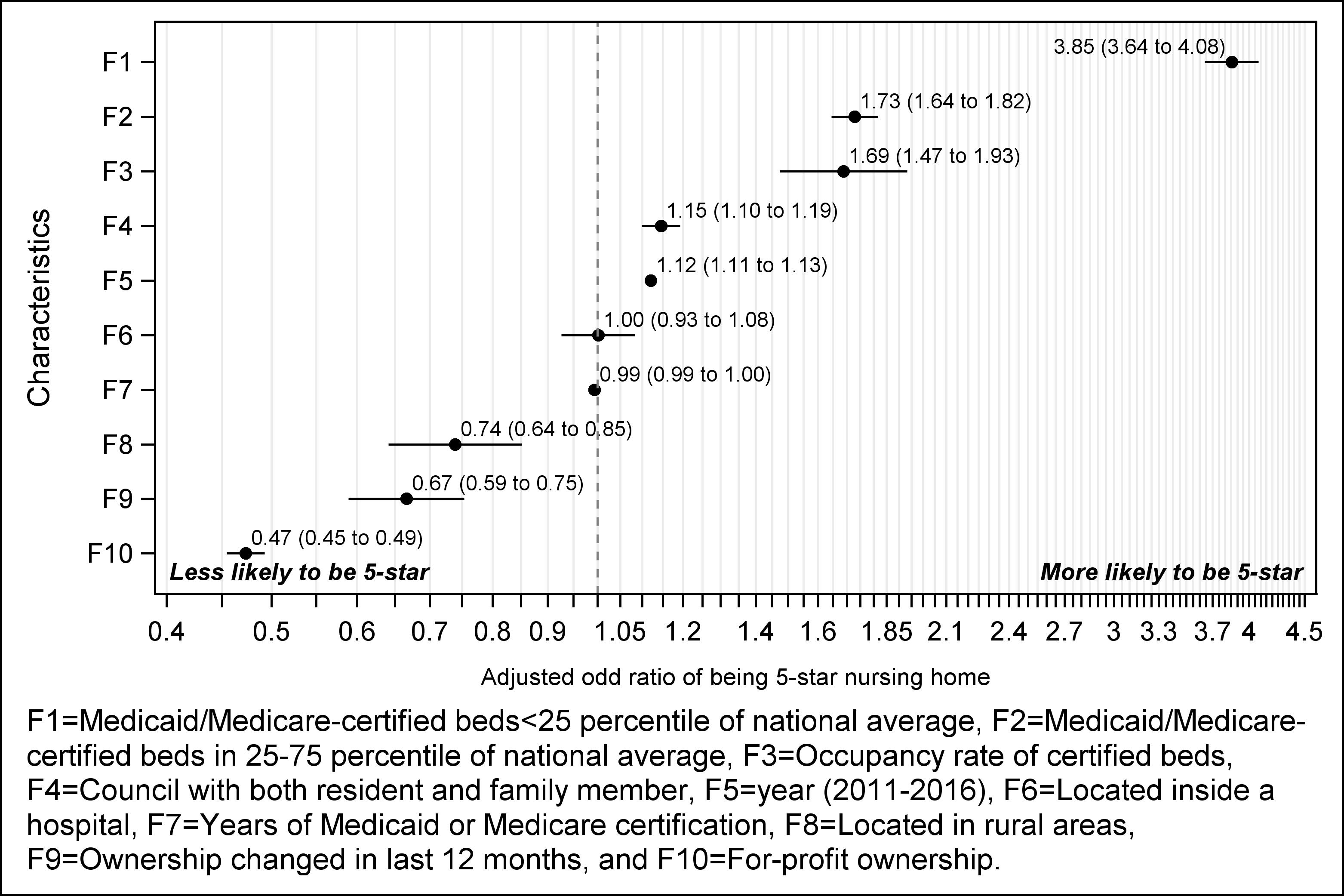
**
